# Unique estimation in EEG analysis by the ordering ICA

**DOI:** 10.1371/journal.pone.0276680

**Published:** 2022-10-24

**Authors:** Yoshitatsu Matsuda, Kazunori Yamaguchi

**Affiliations:** 1 Department of Science and Technology, Seikei University, Tokyo, Japan; 2 Department of General Systems Studies, The University of Tokyo, Tokyo, Japan; University of Bradford, UNITED KINGDOM

## Abstract

Independent Component Analysis (ICA) is a method for solving blind source separation problems. Because ICA only needs weak assumptions to estimate the unknown sources from only the observed signals, it is suitable for Electroencephalography (EEG) analysis. A serious disadvantage of the traditional ICA algorithms is that their results often fluctuate and do not converge to the unique and globally optimal solution at each run. It is because there are many local optima and permutation ambiguities. We have recently proposed a new ICA algorithm named the ordering ICA, a simple extension of Fast ICA. The ordering ICA is theoretically guaranteed to extract the independent components in the unique order and avoids the local optima in practice. This paper investigated the usefulness of the ordering ICA in EEG analysis. Experiments showed that the ordering ICA could give unique solutions for the signals with large non-Gaussianity, and the ease of parallelization could reduce computation time.

## 1 Introduction

Electroencephalography (EEG) is a method of monitoring the electrical activity in the brain [1]. As EEG is a non-invasive and low-cost method without imposing a heavy burden on subjects, it is widely used for measuring brain activity. On the other hand, EEG has only a limited number of noisy channels, and its observed results fluctuate according to the subjects and trials. Therefore, EEG needs advanced and robust techniques for extracting the essential components from such noisy and limited signals. Independent component analysis (ICA) is a widely used technique for analyzing EEG signals [2]. One of the most significant advantages of ICA is its versatility. ICA needs only a simple assumption on the model generating observed signals (the linear mixture of non-Gaussian sources). Therefore, ICA is suitable for EEG analysis where an unknown mixture model changes according to the subjects and trials. An ICA-based analysis tool named EEGLAB has been widely used [3]. One disadvantage of ICA is that it often generates a different solution at each run for the same dataset, unlike principal component analysis (PCA). It reduces the robustness of the analysis. This indeterminateness is caused by the non-linearity of the objective functions of ICA, which makes ICA have many local minima. As non-linearity is essential to ICA, it is difficult for the previous ICA methods to solve this problem.

Recently, we proposed a new ICA method named the ordering ICA [4]. The ordering ICA is theoretically guaranteed to find the unique solution of ICA. Though it was much slower than the previous ICA methods, the paper suggested that parallel processing could accelerate the ordering ICA. This paper constructs a parallel implementation on multi-core CPUs of the ordering ICA and applies it to EEG analysis. The experimental results show that our implementation can extract the essential components more robustly than the previous methods with modest computation time.

This paper is organized as follows. In Section 2, ICA and the ordering ICA are briefly explained. In Section 3, the parallel implementation of the ordering ICA is detailed. Section 4 shows the experimental results of our implementation in EEG analysis. Lastly, we conclude this paper in Section 5.

## 2 Background

### 2.1 Independent component analysis and EEGLAB

Generally, ICA is used for extracting unknown sources from observed signals in an unsupervised manner [5, 6]. ICA assumes the linear mixture model ***X*** = ***A*** ***S***, where ***X*** = (*x*_*im*_) and ***S*** = (*s*_*im*_) (*N* × *M* matrices) are the observed signals and the unknown sources, respectively. ***A*** = (*a*_*ij*_) (an *N*×*N* matrix) is the unknown mixing matrix. *N* and *M* are the dimension of signals and the sample size, respectively. ICA extracts the unknown components in ***A*** so that the sources in ***S*** are statistically independent as much as possible. ICA is solved as an optimization problem, where the objective function is based on some higher-order statistics such as kurtosis. The objective function is derived from an assumption that each source is given independently according to a non-Gaussian distribution. It has been well known that ICA is quite useful for analyzing EEG signals from brain activity. EEGLAB is the most widely used tool implemented in MATLAB [3].

There are many methods of ICA according to the choice of the objective function and the optimization algorithm. The three representative ICA methods are employed in EEGLAB: the extended InfoMax [7] (referred to as InfoMax), Fast ICA [8], and JADE [9]. Many other methods are variations of the above three methods. The third method, JADE, is not applicable to high-dimensional signals. As the EEG channel is generally more than 60, JADE can not extract the essential components from EEG signals. Therefore, we focus on InfoMax and Fast ICA here. InfoMax employs a stochastic gradient algorithm for optimizing one of two hyperbolic tangent based objective functions depending on the currently estimated source distribution. Though InfoMax can find essential components robustly, the convergence of InfoMax is slow. Fast ICA employs Newton-Raphson method in the optimization of kurtosis-based objective functions. The advantage of Fast ICA is the speed of its convergence. The disadvantage is that the succeeding components tend to fluctuate more largely. One reason is that the usual approach of Fast ICA (called the deflation approach) estimates the components one by one under the orthonormality constraint. The errors accumulated by the successive estimation of components cause the fluctuations. On the other hand, such accumulated errors do not occur in InfoMax because InfoMax estimates all the components simultaneously. InfoMax has been preferred to Fast ICA in EEG analysis because of its robustness.

### 2.2 Ordering ICA

The ordering ICA is our recently-proposed method [4], which is a variation of the deflation approach of Fast ICA. In summary, the ordering ICA finds the globally optimal component at each deflation step by repeating the Newton-Raphson method with many different initializations and selecting the best solution from the multiple candidates. Here, we explain the ordering ICA in brief. See [4] for the details.

First, we employed a novel objective function in the ordering ICA. Let ***w***_*i*_ = (*w*_*i*1_, …, *w*_*iN*_) be the *i*-th row of the separating matrix ***W***, which is expected to be the inverse of the mixing matrix ***A***. ***Y*** = (*y*_*im*_) = ***W*** ***X*** denotes the estimated sources. The objective function of the *i*-th component in the ordering ICA is given as follows:

$$\begin{array}{c} \Phi_{i}=ϒ(\sum_{m}y_{im}^{4}/M-3) \end{array}$$ (1)

where

$$\begin{array}{c} ϒ(\alpha_{i})=\alpha_{i}-2\text{log}(\alpha_{i}/2+1). \end{array}$$ (2)

Note that $\alpha_{i}=\sum_{m}y_{im}^{4}/M-3$ is the estimated kurtosis of the *i*-th row of ***Y*** if the row is normalized. Now, the following theorem holds:

**Theorem 1** *We assume that* ***S*** *consists of normalized independent sources and does not include any uniform Bernoulli variable. We also assume that* ***X*** *is given by an invertible linear ICA model* ***X*** = ***AS*** *in the real domain. Then, all the non-Gaussian sources are extracted in descending order of* *Υ*(*κ*_*i*_) *if each* Φ_*i*_ *is globally maximized subject to* ∑_*m*_ *y*_*im*_ = 0 *and* ∑_*m*_ *y*_*im*_ *y*_*jm*_/*M* = *δ*_*ij*_ *for* *j* ≤ *i* *(the Gram-Schmidt orthonormalization), where* *κ*_*i*_ *is the true kurtosis of the* *i-th row of* ***S***.

In other words, by globally maximizing Φ_*i*_ with respect to ***w***_*i*_ one by one for each *i* in the Gram-Schmidt orthonormalization, the true separating matrix ***W*** (and the true mixing matrix ***A*** = ***W***^−1^) can be estimated uniquely if the assumptions are satisfied. *κ*_*i*_ can be estimated by *α*_*i*_ in practice.

Next, the algorithm for maximizing Φ_*i*_ in the Gram-Schmidt orthonormalization is described. It is easily proved from the convexity of *Υ*(*α*_*i*_) that the global maximum of Φ_*i*_ = *Υ*(*α*_*i*_) is one of local maxima and minima of *α*_*i*_. Therefore, we can utilize the deflation approach of the Fast ICA algorithm on the kurtosis [8]. As it finds a local minimum or a local maximum in each run by the Newton-Raphson method, we can find the global maximum of Φ_*i*_ without fail if the starting point of each run is sampled sufficiently densely from the entire space of ***w***_*i*_. Actually, by using Fast ICA to generate many candidates with different random initializations and selecting the best result with the highest Φ_*i*_, the global maximum is expected to be found in most cases. This algorithm estimates ***W*** from given ***X*** and is called “the ordering ICA” (Algorithm 1). Though the original ordering ICA [4] can estimate the number of independent components adaptively, we fix the number of independent components as *N* in this paper.

**Algorithm 1** Ordering ICA

**Require:** ***X***: whitened observed signals. *L*, *K*, *ϵ*: hyper-parameters.

**Ensure:** ***W***: separating matrix.

 1: Initialize ***W*** to the empty matrix

 2: *i* ← 1

 3: **while** *i* ≤ *N* **do**

 4:  **if** *i* > 1 **then**

 5:   ***E*** ← ***W***^⊺^***W***

 6:  **else**

 7:   **E** ← 0

 8:  **else if**

 9:  Initialize randomly *L* different candidates $w_{i}^{l}$

 10:  **for all** *l* ∈ {1, …, *L*} **do**

 11:   ${\hat{w}}_{il}$, ***Y*** ← FastICA ($w_{i}^{l}$, ***X***, ***E***)

 12:   $\alpha_{il}\leftarrow \sum_{m}y_{m}^{4}/M-3$

 13:  **end for**

 14:  *p* ← argmax_*l*_ *Υ*(*α*_*il*_)

 15:  Concatenate ${\hat{w}}_{ip}^{⊺}$ to ***W*** as the last row

 16:  *i* ← *i* + 1

 17: **end while**

 18: **function** FastICA(***w***, ***X***, ***E***)

 19: ***w*** ← ***w*** − ***E*** ***w***

 20: $w\leftarrow w/\sqrt{w^{⊺}w}$

 21: *t* ← 0

 22:  **repeat**

 23:  ***W***_prev_ ← ***w***

 24:  $y\leftarrow w^{⊺}\tilde{X}$

 25:  $w\leftarrow \tilde{X}{\left(y\circ y\circ y\right)}^{⊺}/M-3w$

 26:  ***w*** ← ***w***−***E*** ***w***

 27:  $w\leftarrow w/\sqrt{w^{⊺}w}$

 28:  *t* ← *t* + 1

 29: **untill** *t* < *K* and (||***w*** + ***w***_prev_|| > *ϵ* or ||***w*** − ***w***_prev_|| > *ϵ*)

 30: $y\leftarrow w^{⊺}X$

 31: **return *w***, ***Y***

 32: **end function**

In the ordering ICA, many candidates (the number is denoted by *L*) improve the robustness of the results. Because the total computation time is linear to *L*, the efficiency advantage of Fast ICA is lost if a naive implementation is employed.

## 3 Implementation

Here, we describe the parallel implementation of the ordering ICA. The time-consuming part of ordering ICA is multiple Fast ICA runs with different initializations (the lines 9-13 of Algorithm 1). Because the runs are independent, we can execute the runs in parallel. As the widely used tool EEGLAB was implemented on MATLAB, we implemented this using Parallel Computing Toolbox of MATLAB. We refer to this implementation as the parallel-ordering ICA. See the released code (ParallelOrderingICA.m) for the details at https://github.com/yoshitatsumatsuda/orderingICA/blob/master/ParallelOrderingICA.m.

Notably, this implementation uses a simple convergence condition in the FastICA function. If we run the FastICA function just once, we need to estimate ***w*** with a different initialization if it fails to converge. The parallel-ordering ICA can skip such a reestimation unless all FastICA runs fail to converge.

The hyper-parameter *L* (the number of parallel candidates) is set to be *R* times as large as the number of available cores in the given system. The implemented code is given by L = feature(‘numcores’) * R; in Parallel Computing Toolbox. *R* is the overhead rate. The default value of *R* is set to 1. By using larger *R*, we can increase the number of candidates *L* at the cost of computational overhead. If the convergence threshold *ϵ* is sufficiently small, a slight difference does not have significant effects on the results. So, we just set *ϵ* to 10^−6^. The maximum number *K* of iterations in each FastICA function is set to 30. *K* is relatively small because the convergence failures of some FastICA runs are no problems.

## 4 Experiments on EEG analysis

To verify the usefulness of the proposed method, we used several public EEG datasets and investigated the results from the viewpoint of robustness, the computation time, and the relation between the degree of non-Gaussianity and the success rate of finding a unique solution.

### 4.1 Datasets

**5SUBJECTS** consists of the 10 EEG signals syn{02,05,07,08,10}-s{253,254} downloaded at https://sccn.ucsd.edu/eeglab/download/STUDY5subjects.zip. They were observed in a semantic task with five subjects under two conditions. Each signal has 61 channels, namely, *N* = 61. The sample size *M* depends on the dataset and is about 160,000-190,000.
**STERN** consists of the 39 EEG datasets S{01-13}-{Ignore,Memorize,Probe} downloaded at https://sccn.ucsd.edu/eeglab/download/STUDYstern.zip. They were observed in Sternberg working memory tasks with 13 subjects under three conditions. Each signal has 69-71 channels, namely, *N* = 69, *N* = 70, or *N* = 71. The sample size *M* is about 70,000-100,000 under the Ignore condition (I, for short), about 120,000-180,000 under the Memorize one (M), and about 25,000-35,000 under the Probe one (P).
**TUH** is a dataset of randomly-selected 98 signals from the TUH EEG Corpus downloaded at https://isip.piconepress.com/projects/tuh_eeg/ after registration. The corpus consists of more than 30,000 signals under various conditions, and the number of channels (*N*) is relatively small (about 30). 100 signals were selected randomly from the corpus (v.1.1.0), and two unreadable signals were removed. In the selected 98 signals, *N* is 23-33 and *M* is about 2,000-1,700,000.

### 4.2 Investigation of the robustness and the computation time

To verify the robustness and efficiency of the parallel-ordering ICA, we investigated the averaged fluctuations of the solutions over the different runs and the averaged computation time in 5SUBJECTS and STERN. MATLAB R2022a with Parallel Computing Toolbox was employed. The implementation of the parallel-ordering ICA (called Ordering ICA) in Section 3 is applied directly to each EEG signal. For comparison, we applied the extended InfoMax of EEGLAB 2021.1 (called InfoMax) at https://github.com/sccn/eeglab and the Fast ICA using the kurtosis (FastICA 2.5) at https://research.ics.aalto.fi/ica/fastica/ to the same signals. We did not apply JADE because *N* was too large to employ JADE. We conducted experiments on a 32-core server (dual CPU with 16 cores) with 256GB RAM, where each core is an Intel Xeon 2.1GHz processor.

The fluctuation is measured as follows. Each algorithm (Ordering ICA, InfoMax, and Fast ICA) was applied to each EEG signal from different initializations over 10 runs. The number of runs is denoted by *T* = 10. To remove the permutation ambiguities, ***w***_*i*_’s were ordered by *Υ*(*α*_*i*_), a degree of non-Gaussianity, in all the solutions. Note that the number of ***w***_*i*_’s is often less than the number of channels *N* in Fast ICA. It is because the Fast ICA algorithm often fails to converge if the non-Gaussianity of the signal is low. Now, ***w***_*i*_ in the *p*-th run is denoted by $w_{i}^{p}$ (*p* = 1, …, 10). To estimate the fluctuations of the results for the different runs, we use the divergence *δ*(*i*, *p*, *q*) between $w_{i}^{p}$ and $w_{i}^{q}$, defined as

$$\begin{array}{c} \delta (i,p,q)=1-|\frac{w_{i}^{p}\cdot w_{i}^{q}}{\left\|w_{i}^{p}\|\|w_{i}^{q}\right\|}|. \end{array}$$ (3)

Since the positive or negative sign of ***w***_*i*_ is arbitrary in ICA, the absolute value of the cosine similarity is employed. *δ*(*i*, *p*, *q*) takes the minimum 0 if and only if $w_{i}^{p}$ and $w_{i}^{q}$ have the same or opposite directions. When $w_{i}^{p}$ is orthogonal to $w_{i}^{q}$, *δ*(*i*, *p*, *q*) takes the maximum 1. If $w_{i}^{p}$ (or $w_{i}^{q}$) does not exist due to non-convergence in Fast ICA, *δ*(*i*, *p*, *q*) was set to the maximum 1. Consequently, the fluctuation of ***w***_*i*_ over all the runs is evaluated as the average of *δ*(*i*, *p*, *q*), defined as

$$\begin{array}{c} \bar{\delta }(i)=\frac{\sum_{p=1}^{T}\sum_{q>p}^{T}\delta (i,p,q)}{T(T-1)/2}. \end{array}$$ (4)

**Fig 1:**
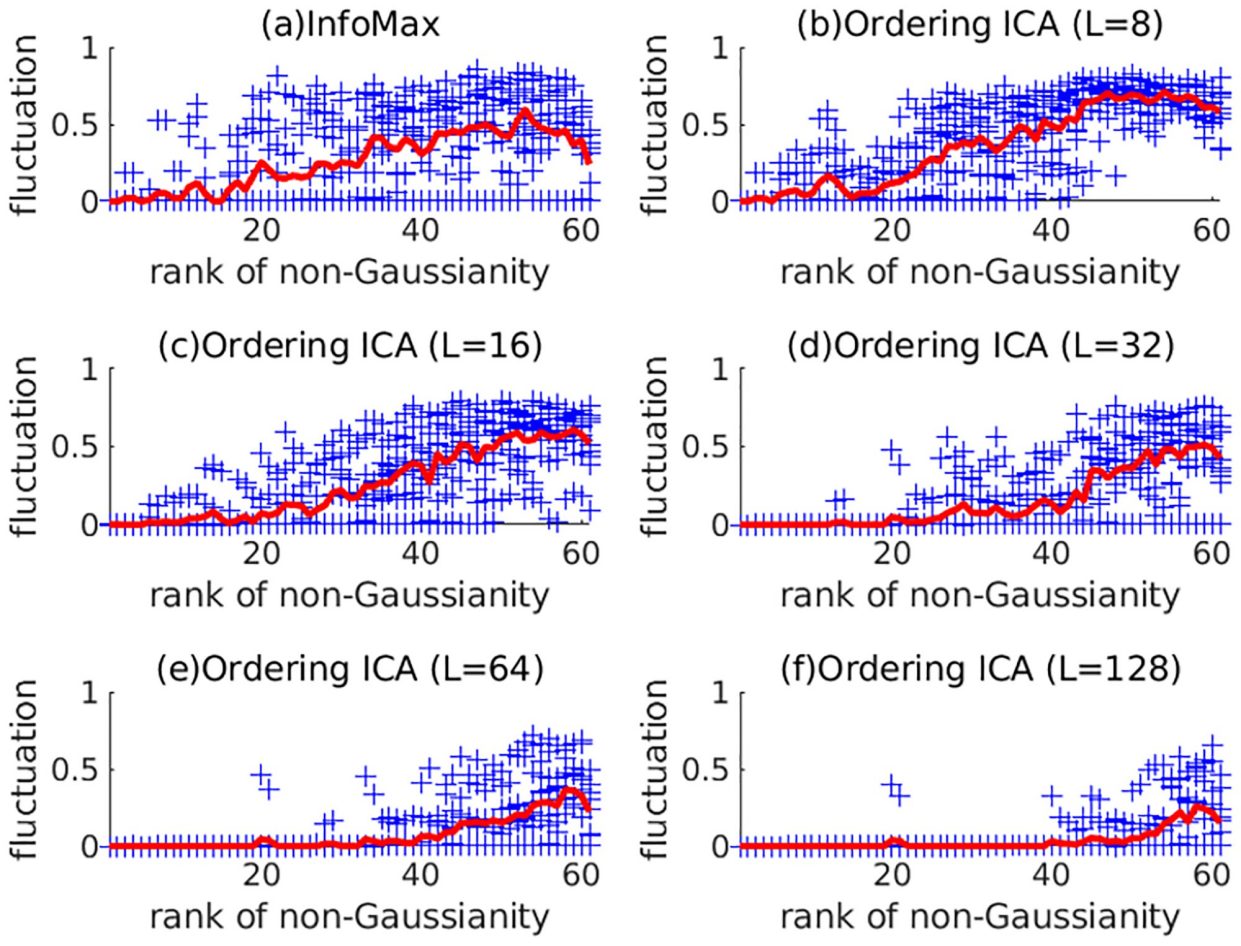
Fluctuation of solutions of ICA in EEG analysis by InfoMax and Ordering ICA with *L* = 8, 16, 32, 64, and 128 for 5SUBJECTS. Each point (denoted by ‘+’) corresponds to the degree of fluctuations measured as $\bar{\delta }(i)$ in each EEG signal. The horizontal index is sorted by the rank of non-Gaussianity of the estimated components. The solid curve shows the average of the fluctuations over the 10 EEG signals for each index.

**Fig 2:**
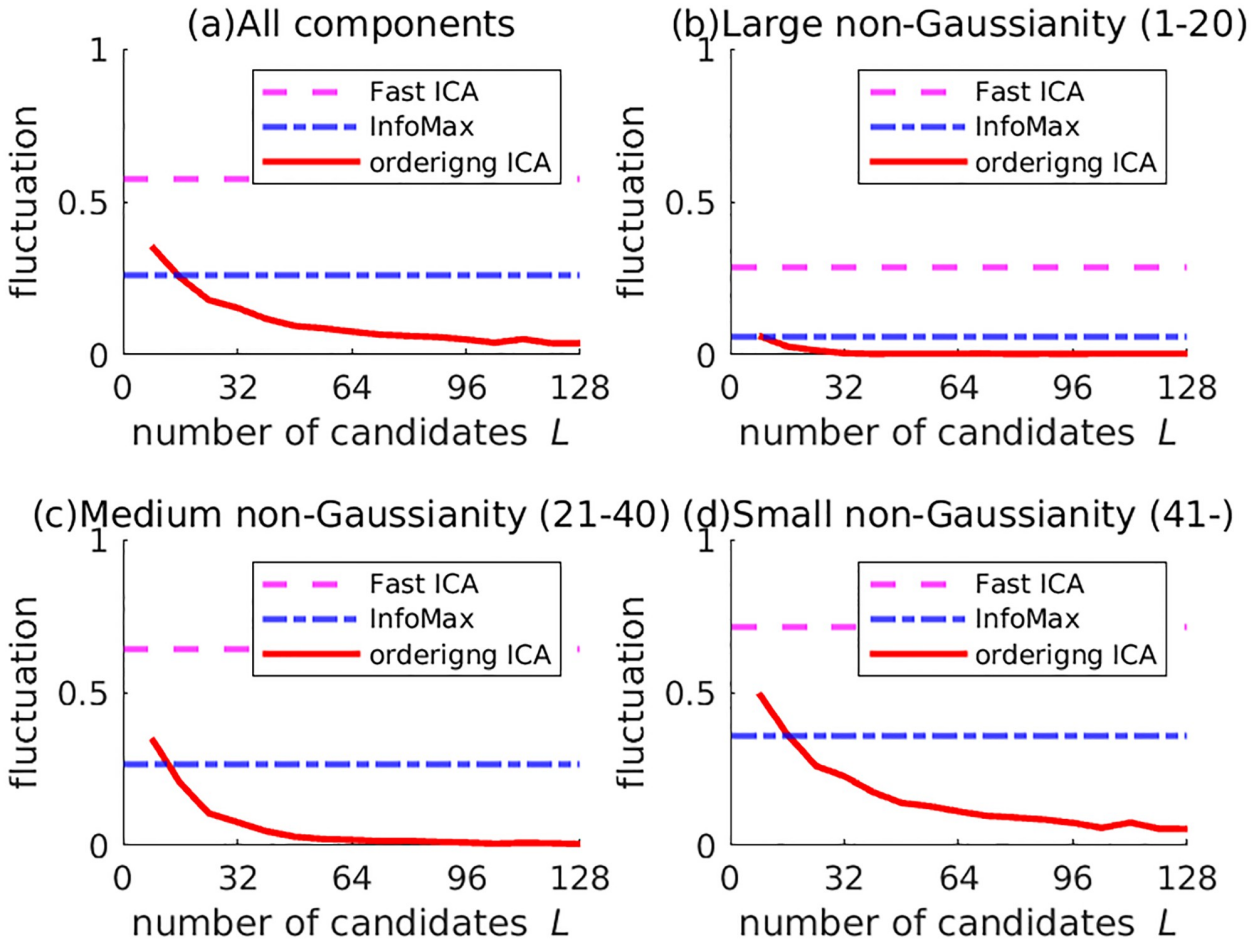
Relation between the number of candidates and the averaged fluctuation over the components for 5SUBJECTS. The solid curve shows the averaged fluctuation over a group of components (all, with large non-Gaussianity (the first-20th ranks), with medium non-Gaussianity (the 21st-40th ranks), with small non-Gaussianity (the rests)). For comparison, the averaged fluctuations of Fast ICA and InfoMax are also shown by the horizontal lines.

**Fig 3:**
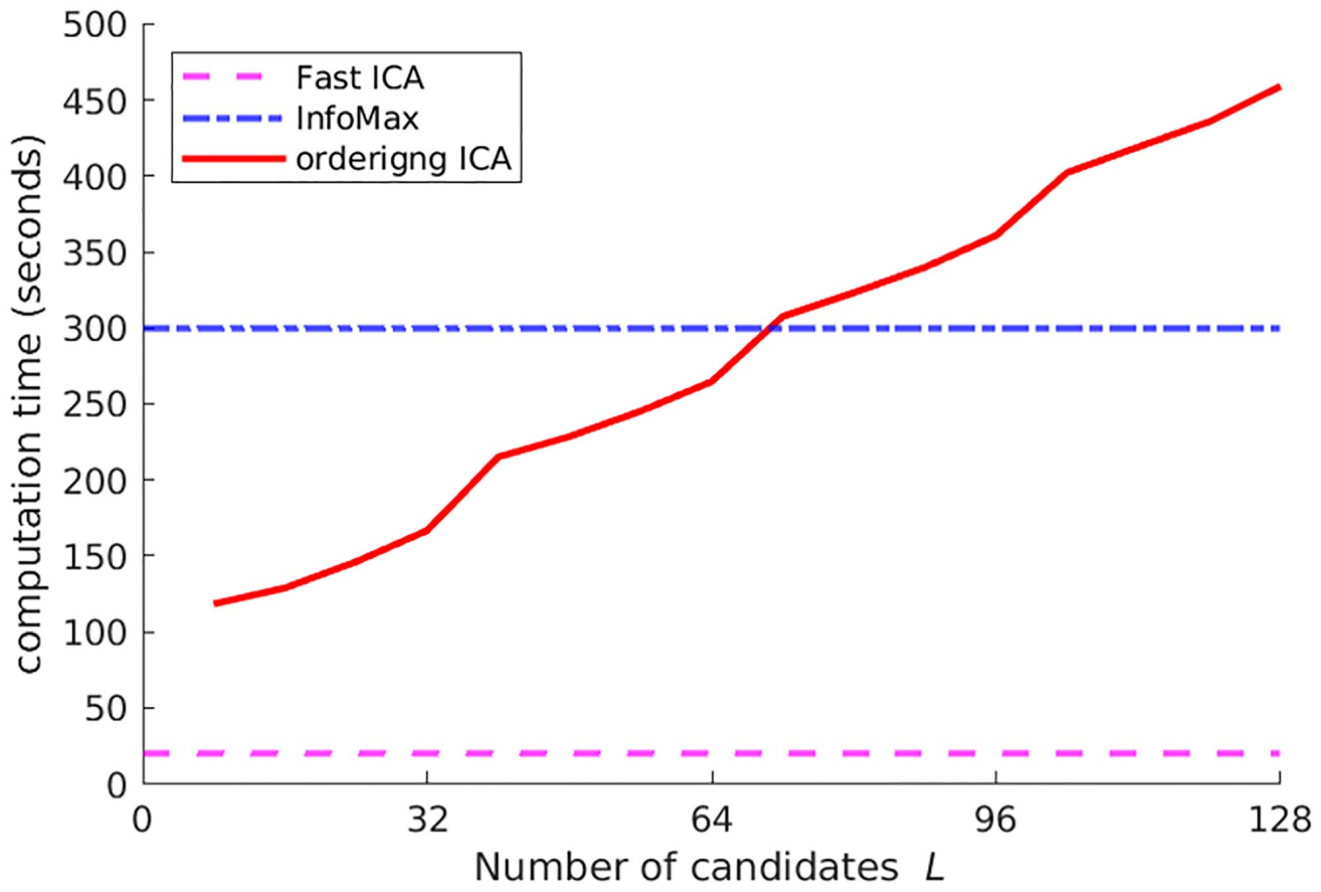
Computation time of Ordering ICA for 5SUBJECTS. The solid curve shows the averaged total computation time over all the signals by the number of candidates *L*. For comparison, the horizontal lines show the averaged computation time of Fast ICA and InfoMax.

First, the experimental results on 5SUBJECTS are shown. The number of candidates *L* in Ordering ICA was set from 8 to 128 by increments of 8. Using the 32-core server, the overhead rate *R* for the 32-core server was set from 0.25 to 4 by increments of 0.25. Fig 1 shows the plot of $\bar{\delta }(i)$ of the 10 signals by InfoMax and Ordering ICA with *L* = 8, 16, 32, 64, and 128 only. For *L* = 8 and *L* = 16, the Ordering ICA results are not so different from InfoMax. For *L* = 32, the fluctuations of the top-ranked 20 components (with relatively large non-Gaussianity) are quite small. For *L* = 128, the fluctuations of the top-ranked 40 components are quite small. Fig 2 clearly shows the relations between the number of candidates *L* and the averaged fluctuation over the components. The fluctuations are averaged over the four different groups of the components: all, the top-ranked 20 ones with large non-Gaussianity, the medium-ranked 20 ones, and the rest ones with small non-Gaussianity. The averaged fluctuation of Ordering ICA is smaller than that of Fast ICA and InfoMax around *L* = 16 in every group of components. Regarding the top-ranked 20 components, the averaged fluctuation of Ordering ICA is near 0 around *L* = 32. Regarding the medium-ranked components, it is near 0 around *L* = 64. Though the averaged fluctuation over the rest components does not converge to 0, it decreases as *L* increases. Fig 3 shows the relation between the computation time of Ordering ICA and the number of candidates *L* in comparison with Fast ICA and InfoMax. Though the computation time is approximately linear to *L*, it is slightly shorter if *L* is a multiple of the number of cores 32. It shows that an integral overhead rate *R* is efficient. It also shows that the computation time of Ordering ICA is shorter than that of InfoMax if *L* is less than 64. Even for *L* = 128, the computation time of Ordering ICA is shorter than the double of that of InfoMax.

**Fig 4:**
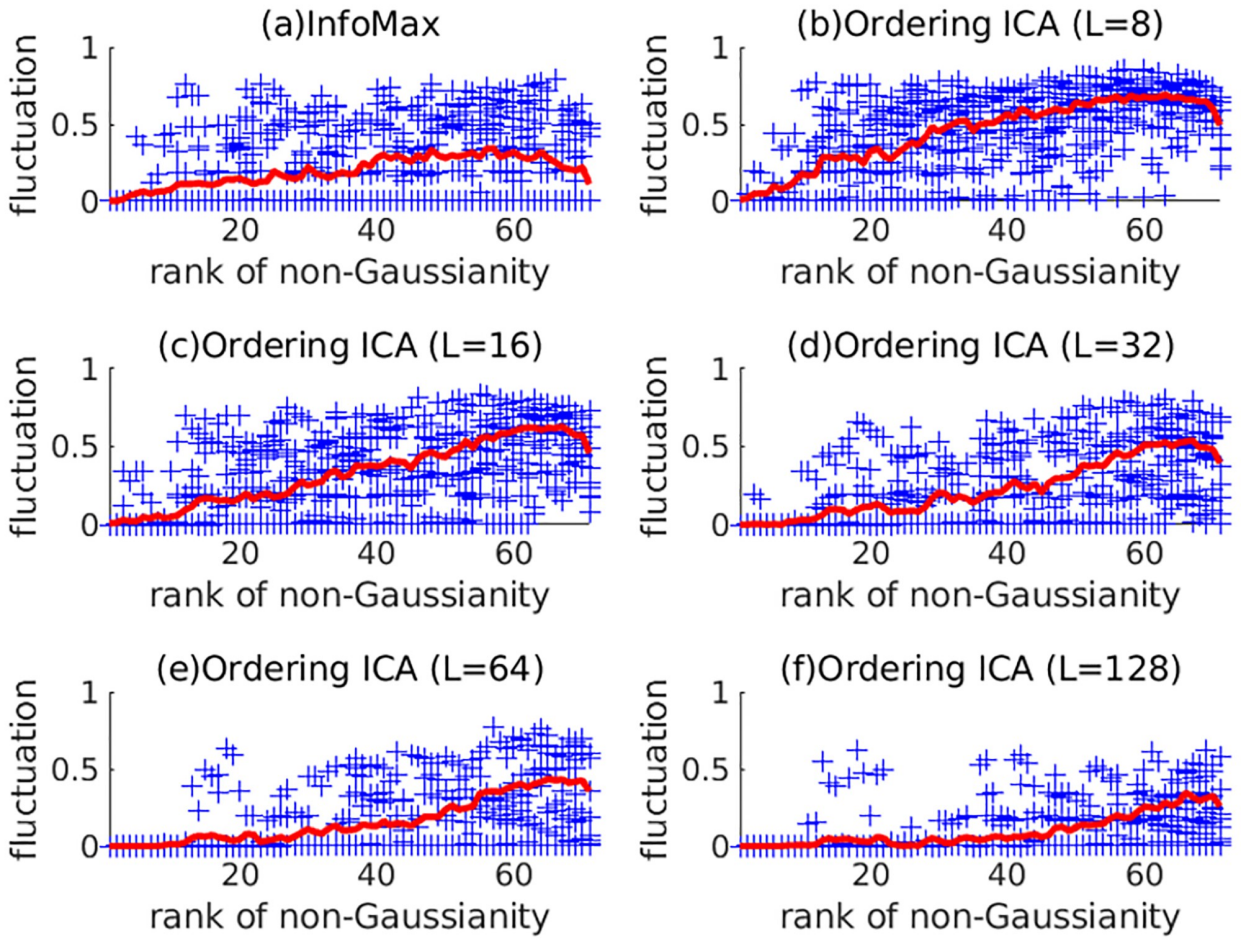
Fluctuation of solutions of ICA in EEG analysis by InfoMax and Ordering ICA with *L* = 8, 16, 32, 64, and 128 for STERN. The points (denoted by ‘+’) and the curves are displayed in the same way as in Fig 1 for the 39 EEG signals of STERN.

**Fig 5:**
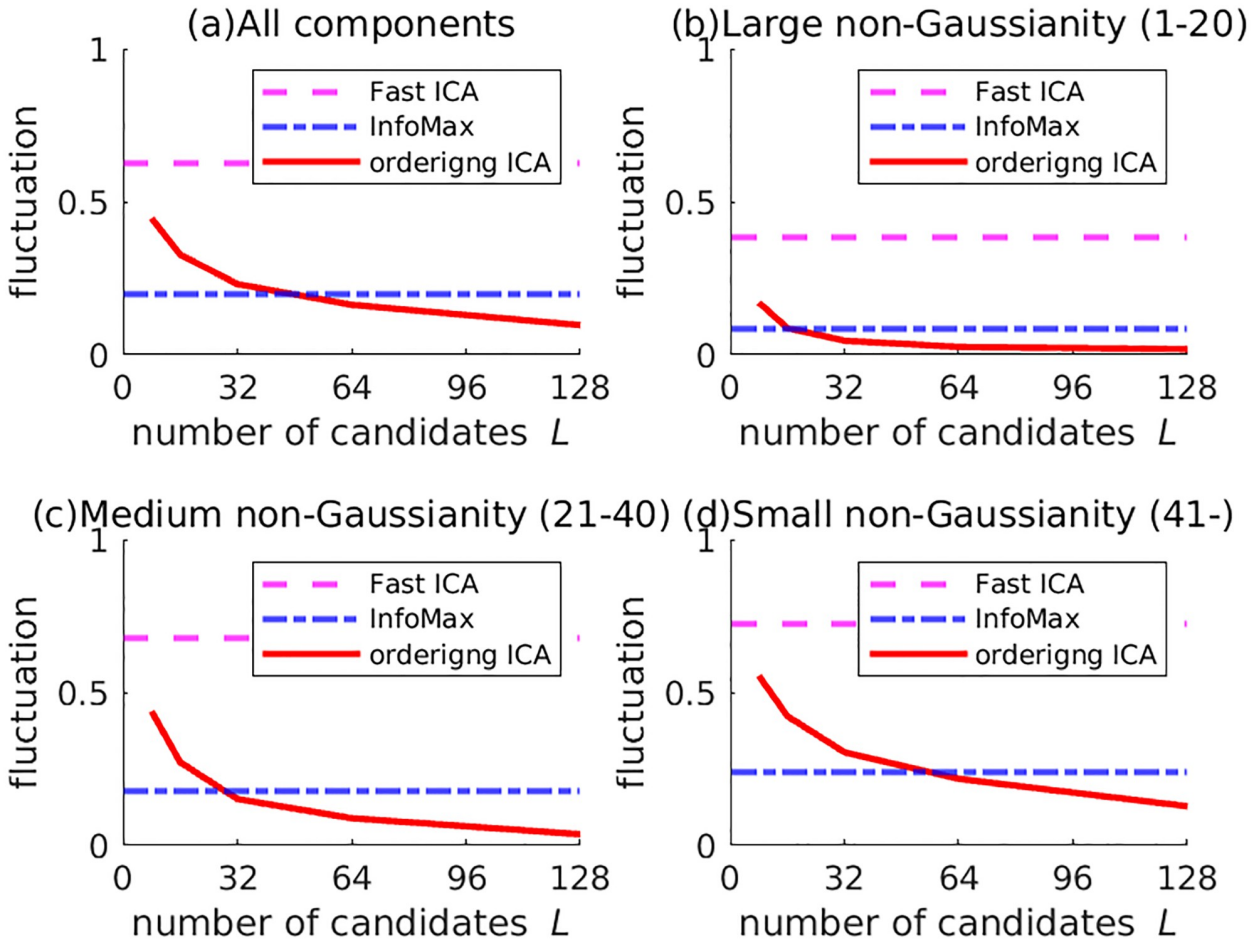
Relation between the number of candidates and the averaged fluctuation over the components for STERN. The curves and the horizontal lines are displayed in the same way as in Fig 2.

**Fig 6:**
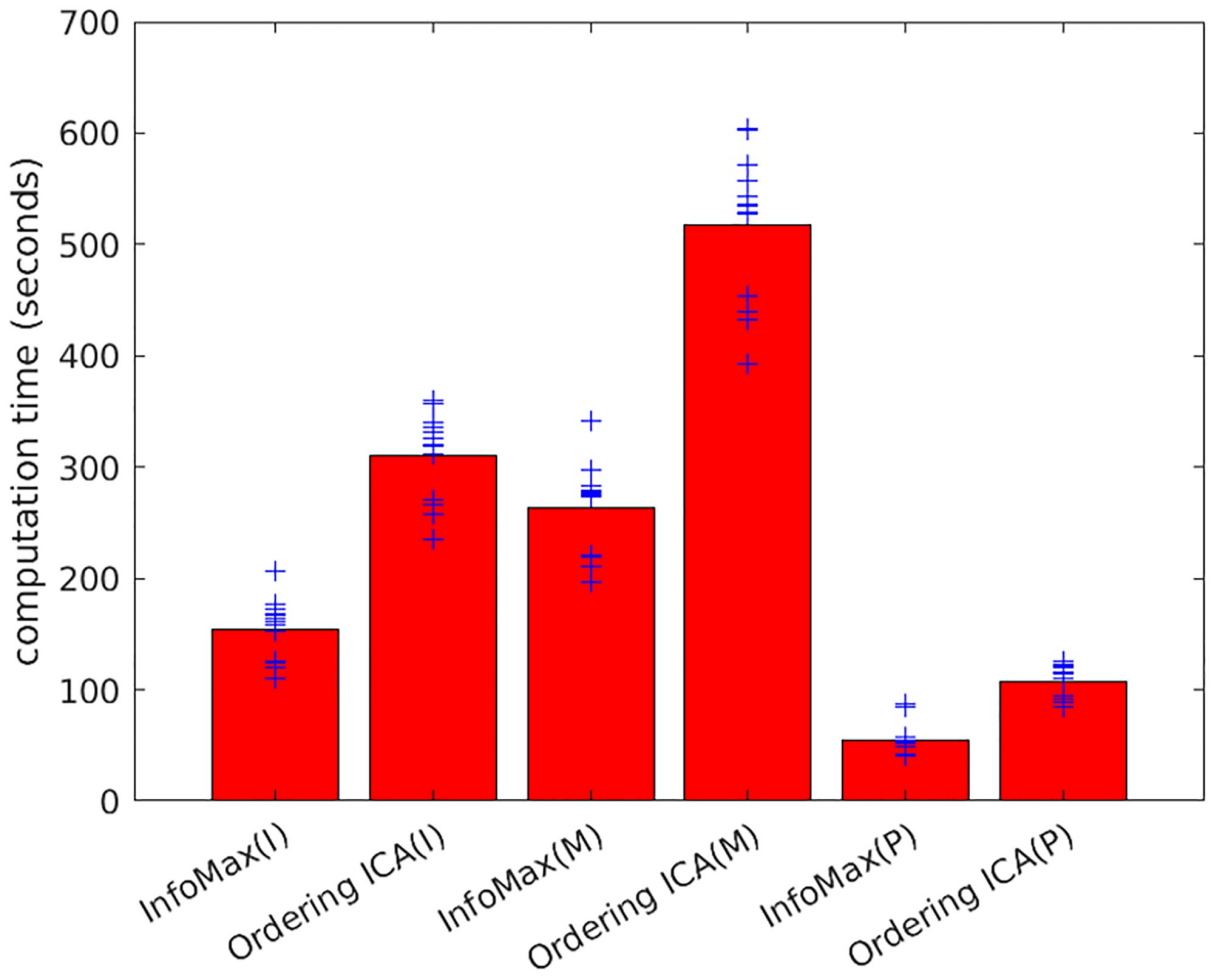
Comparison of computation time of InfoMax and Ordering ICA (*L* = 128). Each point (denoted by ‘+’) shows the averaged computation time over the runs in each signal of STERN. The results are shown under STERN’s three different experimental conditions (I, M, P). The bar shows the computation time averaged over the 13 signals of each condition.

Next, the experimental results on STERN are shown. The number of candidates *L* in Ordering ICA was set to 8, 16, 32, 64, or 128 only. The intermediate settings (e.g. *L* = 24 and *L* = 40) were not carried out to save experimental resources. Except for this saving, the experimental results are shown similarly to 5SUBJECTS. If no corresponding channel exists for *N* < 71, the average was calculated over only the existing data. Fig 4 shows the plot of $\bar{\delta }(i)$ of the 39 signals by InfoMax and Ordering ICA with *L* = 8, 16, 32, 64, and 128. Fig 5 shows the relations between the number of candidates *L*(= 8, 16, 32, 64, 128) and the averaged fluctuation over the components. Note that intermediate numbers of candidates (e.g. *L* = 24) were omitted as opposed to Fig 2. They show that the results of Ordering ICA in STERN are slightly inferior to those in 5SUBJECTS probably because the estimation of the components in STERN is harder than that in 5SUBJECT. The signals of STERN have more channels than those of 5SUBJECTS and the sample size of STERN varies widely. Nevertheless, Ordering ICA is superior to InfoMax around *L* = 64. For *L* = 128, the fluctuation of the top-ranked 20 components and that of the medium-ranked components are near 0. Fig 6 shows the computation time of InfoMax and Ordering ICA (*L* = 128) under STERN’s three conditions (I, M, and P). The sample size *M* is around 100,000 for the condition I, around 150,000 for M, and around 30,000 for P. It shows that the computation time of Ordering ICA with *L* = 128 is at most twice as long as that of InfoMax irrespective of the sample size.

In summary, the ordering ICA algorithm with *L* = 64 is always superior to the widely used InfoMax algorithm from the viewpoint of robustness (measured by the averaged fluctuation). In addition, the computation time of the parallel-ordering ICA algorithm with even *L* = 128 is at most twice as long as that of InfoMax when a 32-core server can be utilized. Though the computation time is roughly linear to *L*, we can reduce the linear factor by using more cores. These results verify the usefulness of the proposed parallel implementation of the parallel-ordering ICA in EEG analysis.

### 4.3 Relation between non-Gaussianity and success rate of finding unique solution

As shown in the preceding section, the parallel-ordering ICA algorithm can find a unique solution in many cases for the components with large non-Gaussianity. However, it is not guaranteed to succeed in all cases because it is a randomized algorithm. Here, the success rate is evaluated empirically. We also discuss the failure rate of the parallel ordering ICA algorithm and the appropriate choice of the number of candidates *L*. In addition to 5SUBJECTS and STERN, TUH is employed as a dataset to verify the versatility for various EEG signals.

The success rate of finding the unique solution of a component was empirically evaluated as follows. Ordering ICA with the largest number of candidates (*L* = *L*_max_) is carried out for each EEG signal in a single run. Let $w_{i}^{k}$ be the *k*-th candidate of the *i*-th components. The estimated *i*-th components $w_{i}^{\hat{k}}$ (selected from all the candidates $w_{i}^{k}$ (*k* = 1, ⋯, *L*_max_)) is assumed to be the globally optimal and unique solution. The success rate can be evaluated as the rate of candidates sufficiently near the unique solution $w_{i}^{\hat{k}}$. The set of such candidates is defined as

$$\begin{array}{c} \Xi =\{w_{i}^{k}|\delta \left(i,k,\hat{k}\right)<\epsilon \}, \end{array}$$ (5)

where $\delta (i,k,\hat{k})$ is the divergence between $w_{i}^{k}$ and $w_{i}^{\hat{k}}$ defined by Eq 3. Then, the success rate *μ*_*i*_ of the *i*-th component is evaluated as

$$\begin{array}{c} {\bar{\mu }}_{i}=\frac{\#\Xi }{L_{\text{max}}}, \end{array}$$ (6)

where #Ξ is the size of the set Ξ. In this paper, *L*_max_ and *ϵ* were set to 1000 and 0.001, respectively. Note that the minimum of ${\bar{\mu }}_{i}$ is 1/*L*_max_. If every candidate of a component diverges, the component is neglected in the following analysis.

**Fig 7:**
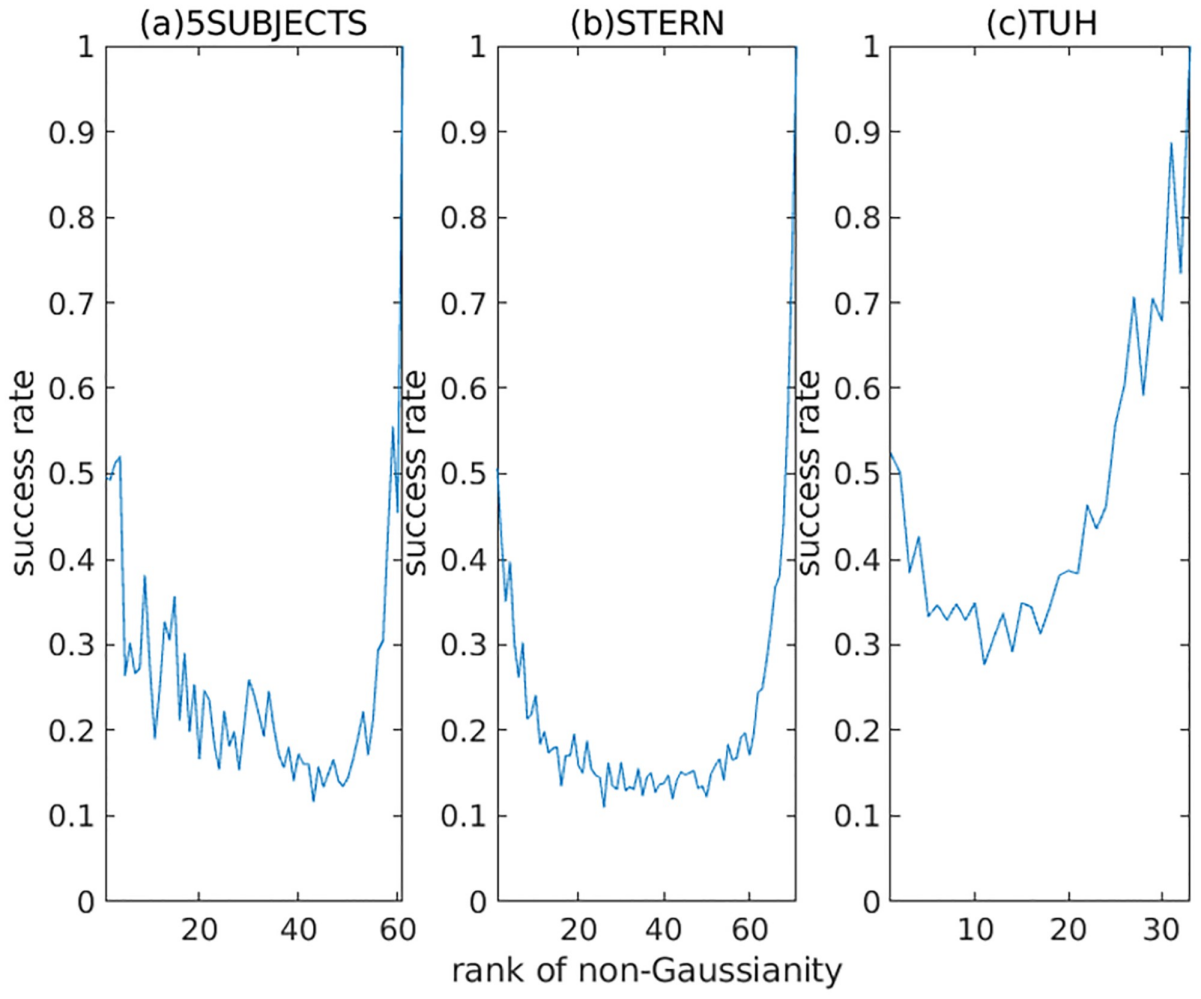
Averaged success rate for 5SUBJECTS, STERN, and TUH. The horizontal index is sorted by the rank of non-Gaussianity of the estimated components. The solid curves show the averages of success rates of finding a unique solution over the signals of each dataset.

Fig 7 shows the averaged success rate of each component (sorted by non-Gaussianity) for 5SUBJECTS, STERN, and TUH. Here, TUH is higher than 5SUBJECTS and STERN, and 5SUBJECTS is slightly higher than STERN. The difficulty of analyzing each dataset is probably proportional to the number of channels. It shows that TUH is easier to solve than 5SUBJECTS and STERN. Fig 7 also shows that the success rate decreases as the non-Gaussianity decreases for the upper-ranked components. On the other hand, the success rate tends to increase as the non-Gaussianity decreases for the lower-ranked components. It is probably because the degree of freedom in the deflation approach is lower for the lower-ranked components. For example, the solution of the last component is necessarily unique (namely, ${\bar{\mu }}_{i}=1$) in a single run.

**Fig 8:**
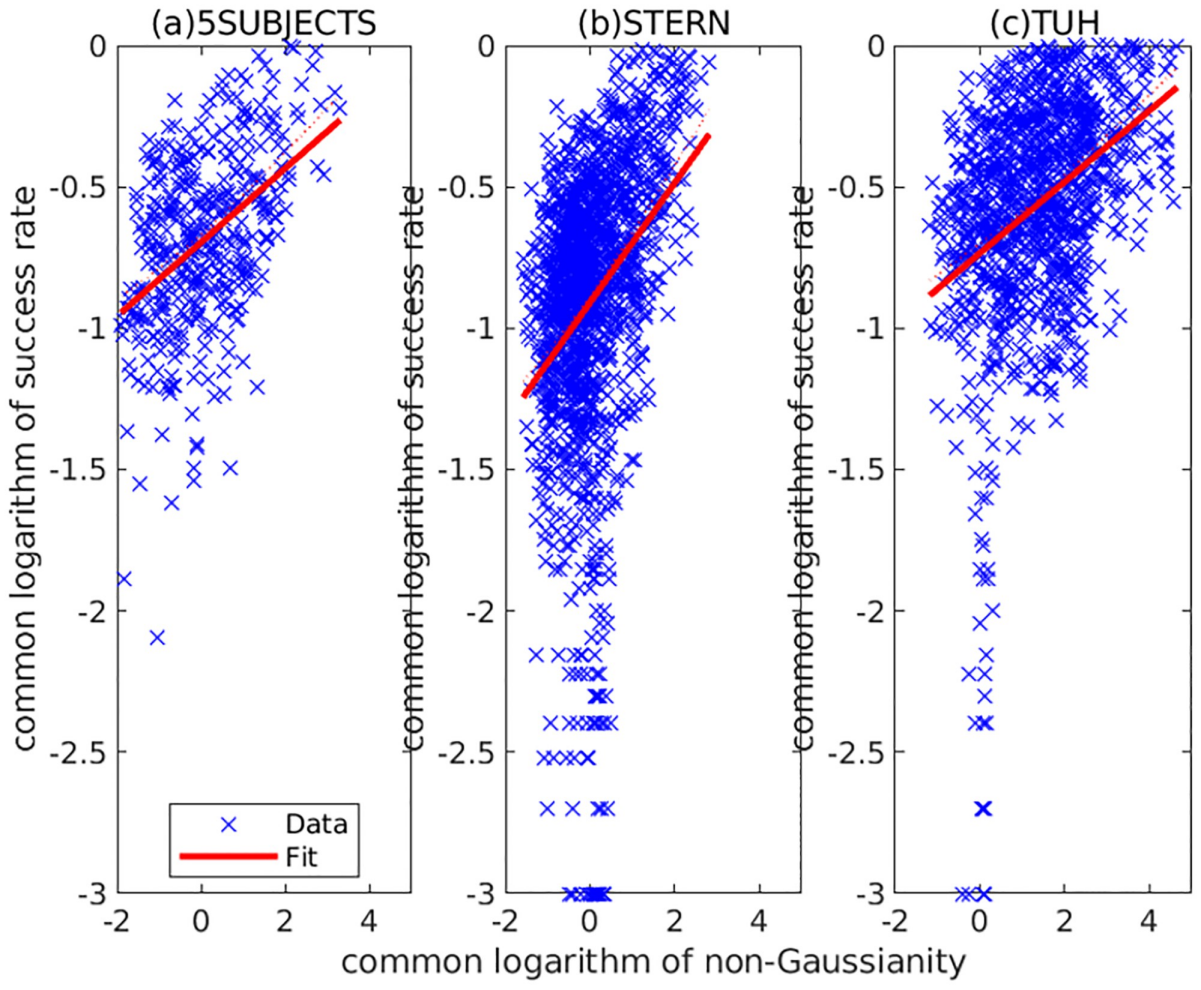
Relation between non-Gaussianity and success rate in upper-ranked components for 5SUBJECTS (upper 40), STERN (upper 40), and TUH (upper 15). Each cross corresponds to $\left({\text{log}}_{10}ϒ\left(\alpha_{i}\right),{\text{log}}_{10}{\bar{\mu }}_{i}\right)$ of a component. The solid line shows the estimated linear regression model ${\text{log}}_{10}\mu_{i}={\hat{\beta }}_{1}+{\hat{\beta }}_{2}{\text{log}}_{10}ϒ\left(\alpha_{i}\right)$. $\left({\hat{\beta }}_{1},{\hat{\beta }}_{2}\right)$ was estimated as (−0.70, 0.20) for 5SUBJECTS, (−0.91, 0.12) for STERN, and (−0.73, 0.18) for TUH.

Fig 8 shows the relation between the non-Gaussianity (measured by log_10_ *Υ*(*α*_*i*_) of Eq 2) and the success rate (measured by ${\text{log}}_{10}{\bar{\mu }}_{i}$) for all the upper-ranked components of the three datasets: 5SUBJECTS (the upper-ranked 40 components), STERN (the upper-ranked 40 components), and TUH (the upper-ranked 15 components). It shows that the relation between log_10_ *Υ*(*α*_*i*_) and ${\text{log}}_{10}{\bar{\mu }}_{i}$ is approximately linear. The worst success rate is estimated by

$$\begin{array}{c} {\hat{\mu }}_{i}≃0.12ϒ{\left(\alpha_{i}\right)}^{0.21} \end{array}$$ (7)

using the worst fitting line for STERN. If the preceding components are estimated global optimally and uniquely, the failure rate $\nu_{i}^{\text{each}}$ in each *i*-th component is estimated by

$$\begin{array}{c} \nu_{i}^{\text{each}}={\left(1-\mu_{i}\right)}^{L}. \end{array}$$ (8)

Then, the total failure rate $\nu_{i}^{\text{total}}$ of failing to find a unique solution is given by the following recurrence relation:

$$\begin{array}{c} \nu_{i}^{\text{total}}=\nu_{i-1}^{\text{total}}+\left(1-\nu_{i-1}^{\text{total}}\right)\nu_{i}^{\text{each}}, \end{array}$$ (9)

where $\nu_{0}^{\text{total}}=0$. Therefore, we can approximately evaluate the failure rate of the upper-ranked component by the non-Gaussianity *Υ*(*α*_*i*_).

**Fig 9:**
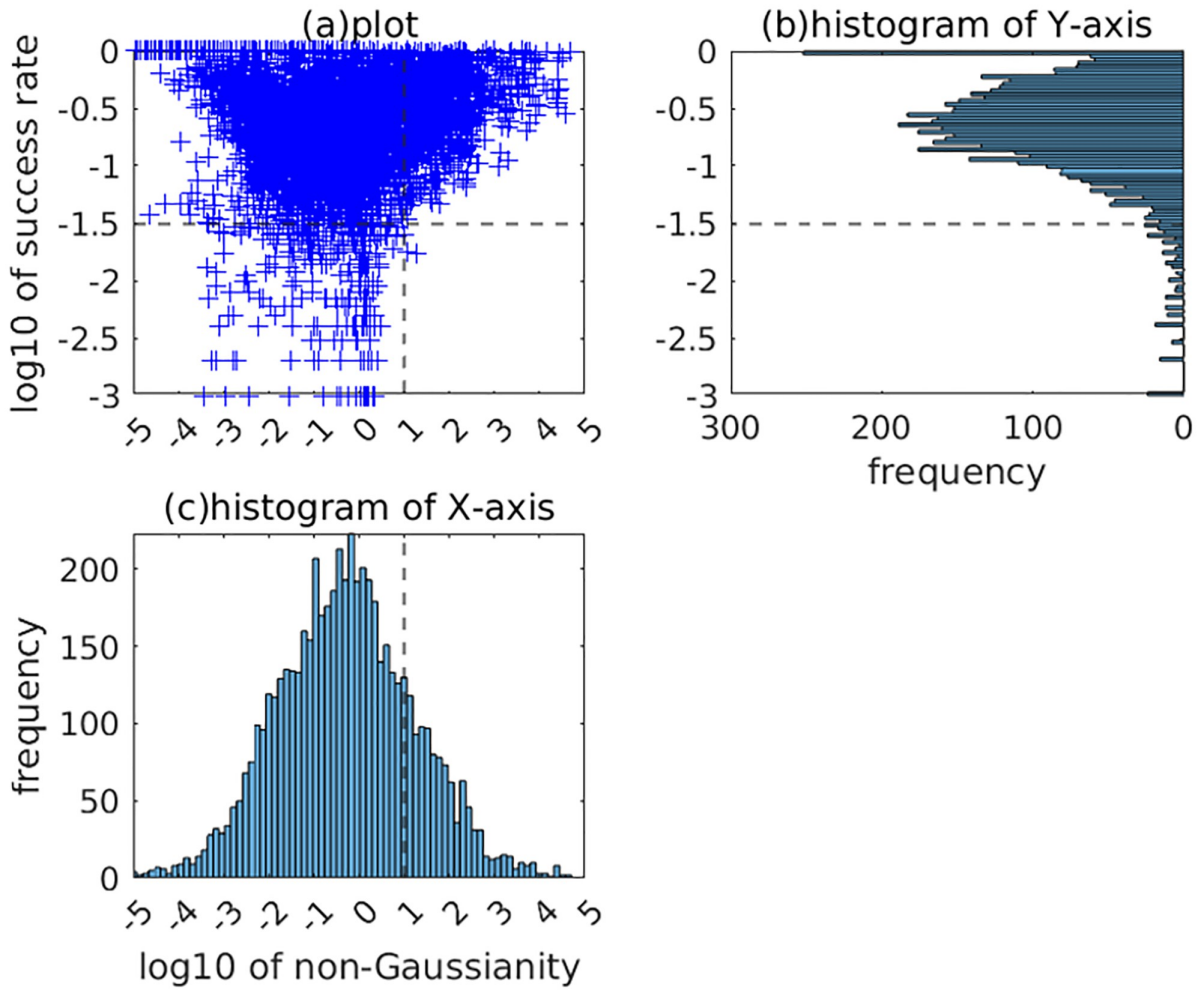
Relation between non-Gaussianity and success rate of all components in all datasets. Each cross in (a) corresponds to $\left({\text{log}}_{10}ϒ\left(\alpha_{i}\right),{\text{log}}_{10}{\bar{\mu }}_{i}\right)$ of a component. (b) and (c) are the histograms of the X-axis and the Y-axis.

Fig 9 plots all the components of all datasets with the non-Gaussianity on the X-axis and the success rate on the Y-axis. It also displays the histograms of each axis. It shows that there are some bounds. For log_10_ *Υ*(*α*_*i*_)>1 (namely, *Υ*(*α*_*i*_)>10), ${\bar{\mu }}_{i}>10^{-1.5}$ holds in almost all cases. Therefore, the failure rate $\nu_{i}^{\text{each}}$ of the components with *Υ*(*α*_*i*_)>10 is bounded from above by (1−10^−1.5^)^*L*^ ≃ 10^−0.0139*L*^. For example, $\nu_{i}^{\text{each}}<0.0164$ for *L* = 128. If we employ a larger *L* (for example, *L* = 256), $\nu_{i}^{\text{each}}$ can be reduced sufficiently ($\nu_{i}^{\text{each}}<3\times 10^{-4}$). The total failure rate also can be reduced ($\nu_{i}^{\text{total}}<i\times 3\times 10^{-4}$) if *Υ*(*α*_*i*_)>10 holds in the top-ranked *i* components. Because the number of components with *Υ*(*α*_*i*_)>10 and the total number of components are 1096 and 5713 in the three datasets, the top 20 percent of the components with large non-Gaussianity are expected to be estimated uniquely for *L* = 256. On the other hand, some components have ${\bar{\mu }}_{i}<10^{-1.5}$ if log_10_ *Υ*(*α*_*i*_)<1. Finding a unique solution for such components is often difficult. For example, the evaluated ${\bar{\mu }}_{i}$ was often the smallest value of 1/1000. It shows that Ordering ICA failed to find a unique solution even for *L* = 1000 in such cases. Because the number of the components with ${\bar{\mu }}_{i}<10^{-1.5}$ is 392 in the three datasets, they are expected to be less than 10 percent of all components. Nevertheless, it should be noted that the total failure rate $\nu_{i}^{\text{total}}$ can be drastically high if the estimation of a preceding component fails.

In summary, it was observed that there was a linear regression model between the logarithm of the non-Gaussianity and that of the success rate. We can estimate the failure rate of finding a unique solution by the model. In addition, the failure rate was low enough to find a unique solution almost certainly by Ordering ICA with an available number of candidates (for example, *L* = 256) if the non-Gaussianity is relatively large (*Υ*(*α*_*i*_)>10).

## 5 Conclusion

This paper proposed the parallel-ordering ICA algorithm. The experimental results on standard EEG datasets verified that our proposed implementation is superior to the widely-used InfoMax of EEGLAB in both stability and efficiency.

We plan to investigate the usefulness of the parallel-ordering ICA algorithm in practical EEG analysis. In addition, we are planning to accelerate the parallel computation by GPUs and distributed systems. Especially, the utilization of the distributed systems may be promising because the parallelization of the ordering ICA is quite simple.
